# A Mycobacterium tuberculosis NBTI DNA Gyrase Inhibitor Is Active against Mycobacterium abscessus

**DOI:** 10.1128/AAC.01514-21

**Published:** 2021-11-17

**Authors:** Uday S. Ganapathy, Rubén González del Río, Mónica Cacho-Izquierdo, Fátima Ortega, Joël Lelièvre, David Barros-Aguirre, Wassihun Wedajo Aragaw, Matthew D. Zimmerman, Marissa Lindman, Véronique Dartois, Martin Gengenbacher, Thomas Dick

**Affiliations:** a Center for Discovery and Innovation, Hackensack Meridian Health, Nutley, New Jersey, USA; b Global Health Pharma Unit, GlaxoSmithKline, Tres Cantos, Madrid, Spain; c Department of Medical Sciences, Hackensack Meridian School of Medicine, Nutley, New Jersey, USA; d Department of Microbiology and Immunology, Georgetown University, Washington, DC, USA

**Keywords:** novel bacterial topoisomerase inhibitor, *Mycobacterium tuberculosis* gyrase inhibitor, EC/11716, nontuberculous mycobacteria, *Mycobacterium abscessus*, DNA gyrase

## Abstract

Fluoroquinolones—the only clinically used DNA gyrase inhibitors—are effective against tuberculosis (TB) but are in limited clinical use for nontuberculous mycobacteria (NTM) lung infections due to intrinsic drug resistance. We sought to test alternative DNA gyrase inhibitors for anti-NTM activity. Mycobacterium tuberculosis gyrase inhibitors (MGIs), a subclass of novel bacterial topoisomerase inhibitors (NBTIs), were recently shown to be active against the tubercle bacillus. Here, we show that the MGI EC/11716 not only has potent anti-tubercular activity but is active against M. abscessus and M. avium *in vitro*. Focusing on M. abscessus, which causes the most difficult to cure NTM disease, we show that EC/11716 is bactericidal, active against drug-tolerant biofilms, and efficacious in a murine model of M. abscessus lung infection. Based on resistant mutant selection experiments, we report a low frequency of resistance to EC/11716 and confirm DNA gyrase as its target. Our findings demonstrate the potential of NBTIs as anti-M. abscessus and possibly broad-spectrum anti-mycobacterial agents.

## INTRODUCTION

While the global incidence of tuberculosis (TB) has declined in recent years, infections by nontuberculous mycobacteria (NTM) are on the rise (1, 2). NTM lung disease is the most common clinical presentation and is primarily caused by members of the M. abscessus and M. avium complexes. Although the two NTM are close relatives of Mycobacterium tuberculosis, these NTM species exhibit differential pathogenesis due to their expression of novel surface lipids, adaptation to both host and environmental niches, and acquisition of novel virulence factors (3). While lung disease is most common, M. abscessus and M. avium infections can also cause severe disseminated disease in immunocompromised individuals (4). In addition, M. abscessus and M. avium demonstrate intrinsic resistance to a broad range of antibiotics (5–7). The current drug regimens for NTM lung disease vary by species and differ from the standard four-drug TB chemotherapy (8–10). M. abscessus presents the most difficult to cure NTM disease. The treatment regimen typically combines a macrolide with a parenterally administered drug (amikacin or imipenem) and either cefoxitin or tigecycline. The potency of macrolides against M. abscessus can be limited by *erm*(41)-mediated inducible drug resistance (11). Nonetheless, macrolides still provide beneficial immunomodulatory effects such as reducing airway secretion to promote mucociliary clearance (12, 13). The need for intravenous drug administration in M. abscessus chemotherapy is another complicating factor not encountered in M. avium treatment, where all drugs can be administered orally. With a cure rate of around 50%, the treatment outcomes for patients with M. abscessus infections remain unsatisfactory (14). Thus, new drugs are needed to curb the rise in NTM infections, including essentially “incurable” M. abscessus lung disease (15, 16).

DNA gyrase is a validated drug target in mycobacteria. This type IIA DNA topoisomerase is a A_2_B_2_ heterotetrameric protein that regulates DNA topology (17). The unwinding of DNA during replication, transcription and recombination introduces positive supercoils into the DNA molecule that, left unaddressed, will impede DNA function (18). This problem is resolved by DNA gyrase, which introduces negative supercoils into DNA (17). To do this, the enzyme generates a DNA double-stranded break, passes a separate segment of double-stranded DNA through the break, and subsequently reseals the DNA molecule (17). The DNA gyrase inhibitor moxifloxacin is used effectively for the treatment of multidrug-resistant TB (19). The fluoroquinolone targets the cleavage-ligation active site of DNA gyrase, creating stalled enzyme-DNA cleavage complexes (20). Conversion of these complexes into permanent double-stranded DNA breaks can kill the bacterium, giving fluoroquinolones bactericidal activity. Moxifloxacin is also in use as a second line drug against certain NTM diseases (10, 21, 22). However, the clinical utility of the fluoroquinolone for the treatment of NTM infections is very limited. Recent reports suggest that clinical isolates from both the M. abscessus and M. avium complexes are mostly resistant to moxifloxacin (23–27). Whereas fluoroquinolone resistance in M. tuberculosis occurs via acquired QRDR (quinolone resistance determining region) mutations in the DNA gyrase encoding *gyrA* and *gyrB* genes (28, 29), a study of 72 moxifloxacin-resistant clinical isolates of M. abscessus and M. avium found no QRDR mutations in these genes (30). These findings suggest that, unlike TB, NTM harbor (undetermined) intrinsic fluoroquinolone resistance mechanisms. Given the limited efficacy of currently used NTM drugs, a more effective DNA gyrase inhibitor would be a welcome addition to NTM treatment regimens (27, 31).

Novel bacterial topoisomerase inhibitors (NBTIs) are a new generation of DNA gyrase inhibitors developed against Gram-positive and -negative bacteria (32–39). Compared to fluoroquinolones, NBTIs have a different structure, consisting of a left-hand side (LHS) portion and a right-hand side (RHS) portion that are linked together by a central unit (CU). NBTIs also target DNA gyrase through a unique mechanism of action. While the RHS of the NBTI scaffold binds to a transient, noncatalytic pocket at the interface of the two GyrA subunits, the LHS intercalates into the DNA midway between the two DNA cleavage sites (36, 40). Through this action, NBTIs stabilize enzyme-DNA cleavage complexes that, unlike fluoroquinolones, generate single-stranded DNA breaks instead of double-stranded ones (40, 41). Mycobacterium tuberculosis gyrase inhibitors (MGIs) are a subclass of NBTIs that were discovered by GlaxoSmithKline (42). MGIs are based on the NBTI scaffold with a 7-substituted-1,5-naphthyridin-2-one in the LHS, an aminopiperidine as the CU, and a monocyclic aromatic ring in the RHS. MGI-resistant M. tuberculosis mutants have mutations in DNA gyrase, establishing the mycobacterial topoisomerase as the target of MGIs (42). Consistent with the mechanism of action and activity of NBTIs, MGIs induce single-stranded DNA breaks and are bactericidal against M. tuberculosis (41, 42). MGIs also retain potency against fluoroquinolone-resistant M. tuberculosis strains (42). For lead MGIs, efficacy in a mouse model of TB infection was demonstrated (42).

**FIG 1:**
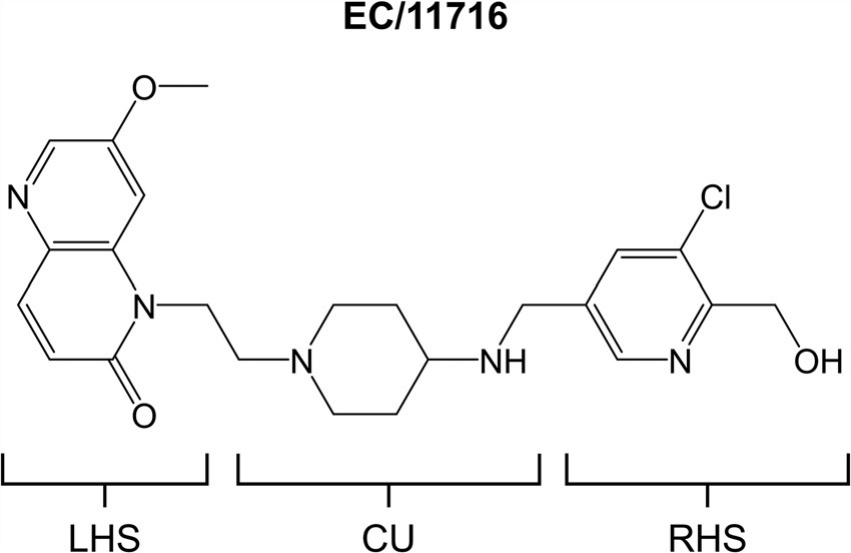
Structure of EC/11716. Left-hand side (LHS), central unit (CU) and right-hand side (RHS) portions of the MGI scaffold are indicated.

To explore the potential of MGIs for the development of anti-NTM drugs, we selected EC/11716 (Fig. 1), a lead MGI with attractive TB activity, and profiled the compound *in vitro* and *in vivo* for its anti-NTM activity. We focused our analyses on M. abscessus, which presents the most drug-resistant NTM lung disease. Our results establish this MGI as a novel lead compound against NTM.

## RESULTS

### TB active EC/11716 is active against M. abscessus and M. avium *in vitro*.

EC/11716 is a lead MGI with potent anti-TB activity (Fig. 1, Table 1). To determine whether this compound is also active against NTM, we first measured its potency against our screening strains M. abscessus Bamboo (subsp. *abscessus*) (43) and M. avium 11 (subsp. *hominissuis*) (44) in Middlebrook 7H9 medium. EC/11716 was active against both mycobacteria (Table 1), suggesting activity against the two major NTM pathogens. NTM lung disease is most commonly caused by members of the M. avium complex, including M. avium and M. intracellulare (10, 45). In addition to its activity against M. avium 11, EC/11716 inhibited the growth of a culture collection reference strain of M. intracellulare (Table 1). Taken together, EC/11716’s ability to target M. tuberculosis, M. abscessus and two representatives of the M. avium complex suggests that this MGI has broad anti-mycobacterial activity. Since M. abscessus lung disease is the most difficult to cure, we focused our subsequent studies of EC/11716 on this mycobacterial species.

**TABLE 1:** Activity of EC/11716 against M. tuberculosis and members of the M. avium *and* M. abscessus complexes^*a*^

		MIC (μM)^*b*^		
**Strain**	**Strain type**	**CLR**	**MXF**	**EC/11716**
M. tuberculosis H37Rv ATCC 27294	Culture collection reference strain	ND^*c*^	0.6	0.38
M. avium complex members				
M. avium 11	Clinical isolate, screening strain	0.30	1.3	1.1
M. intracellulare ATCC 13950	Culture collection reference strain	0.15	0.40	3.3
M. abscessus complex members				
M. abscessus Bamboo	Clinical isolate, screening strain	0.23	4.6	2.5
M. abscessus subsp. *abscessus* ATCC 19977	Culture collection reference strain	0.90	3.6	1.8
M. abscessus subsp. *massiliense* CCUG 48898T	Culture collection reference strain	0.19	8.0	4.7
M. abscessus subsp. *bolletii* CCUG 50184T	Culture collection reference strain	2.5	6.3	4.1
M. abscessus subsp. *abscessus* M9	Clinical isolate	0.73	2.9	2.9
M. abscessus subsp. *abscessus* M199	Clinical isolate	2.7	4.8	3.8
M. abscessus subsp. *abscessus* M337	Clinical isolate	0.90	2.9	2.4
M. abscessus subsp. *abscessus* M404	Clinical isolate	0.20	5.0	2.6
M. abscessus subsp. *abscessus* M422	Clinical isolate	0.65	2.7	1.8
M. abscessus subsp. *bolletii* M232	Clinical isolate	0.95	3.2	2.5
M. abscessus subsp. *bolletii* M506	Clinical isolate	0.28	7.1	5.2
M. abscessus subsp. *massiliense* M111	Clinical isolate	0.24	6.7	4.9
M. abscessus subsp. *abscessus* K21	Clinical isolate, infection model	0.40	7.3	3.0

a MIC values are the mean of two independent experiments.

b CLR, clarithromycin; MXF, moxifloxacin; ND, not determined.

c MIC of rifampin against M. tuberculosis H37Rv was 0.66 μM.

### EC/11716 is active against M. abscessus subspecies and clinical isolates.

Given EC/11716’s activity against M. abscessus Bamboo (Table 1), we asked whether EC/11716 has activity against all three subspecies of the M. abscessus complex (subsp. *abscessus*, subsp. *massiliense*, and subsp. *bolletii*), which are known to exhibit differential antibiotic susceptibility (8, 46). EC/11716 was active against culture collection reference strains for all three subspecies of the complex (Table 1). In addition, EC/11716 maintained comparable activity against a panel of clinical isolates covering the M. abscessus complex (Table 1) (47). EC/11716 also displayed activity against M. abscessus subsp. *abscessus* K21, a clinical isolate used in our M. abscessus mouse infection model (Table 1) (48). These results suggest that EC/11716 retains activity across the M. abscessus complex.

### EC/11716 shows medium independence and is bactericidal against M. abscessus *in vitro*.

A compound’s *in vitro* potency can depend on the composition of the medium and the presence of detergents (49, 50). We therefore remeasured the MIC of EC/11716 in cation-adjusted Mueller-Hinton (CAMH) broth—a standard medium for clinical antibiotic susceptibility testing that has a different carbon source composition from 7H9 and no detergent (51). EC/11716 was active against M. abscessus Bamboo in CAMH. With a MIC of 6.5 μM, activity was slightly weaker compared to 7H9 broth (MIC = 2.5 μM). Thus, the culture medium appears to affect the anti-NTM activity of EC/11716 only moderately.

MGIs have bactericidal activity against M. tuberculosis (42). We asked whether EC/11716 is bactericidal against M. abscessus by determining the MBC (minimum bactericidal concentration; concentration causing a 10-fold reduction in CFU/ml compared to time point 0) of this compound against bacteria growing in culture tubes (52). Clarithromycin inhibited the growth of M. abscessus Bamboo (MIC = 0.28 μM) but had no bactericidal activity (MBC > 100 μM) (Table 2), consistent with the bacteriostatic profile of macrolides against this bacterium (52). In contrast, EC/11716 both inhibited M. abscessus Bamboo growth (MIC = 1.5 μM) and was bactericidal at 2× its MIC (MBC = 3.1 μM) (Table 2). Both the growth inhibition and bactericidal activities of EC/11716 were comparable to those observed for moxifloxacin (Table 2). Consistent with the profile of MGIs against M. tuberculosis (42), our results show that MGIs are also bactericidal against M. abscessus.

**TABLE 2:** Activity profiling of EC/11716 against planktonic, biofilm and nonreplicating M. abscessus Bamboo^*a*^

	MIC (μM)^*b*^			MBC (μM)		
	**CLR**	**MXF**	**EC/11716**	**CLR**	**MXF**	**EC/11716**
Planktonic	0.28	1.9	1.5	>100	3.1	3.1
Biofilm	1.6	6	0.78	>100	25	6.3
Anaerobic, nonreplicating	NA	NA	NA	>100	>100	>100
Nutrient-starved, nonreplicating	NA	NA	NA	>100	>100	>100

a MIC and MBC values are the mean of two independent experiments.

b CLR, clarithromycin; MXF, moxifloxacin; NA, not applicable.

### EC/11716 is effective against M. abscessus biofilms.

M. abscessus forms biofilms that are tolerant to several classes of NTM drugs (52–54). We therefore assessed the activity of EC/11716 in an *in vitro* M. abscessus Bamboo biofilm growth assay (52). The MICs of both clarithromycin and moxifloxacin increased against M. abscessus biofilms compared to planktonic bacteria (Table 2). In contrast, the MIC of EC/11716 was unchanged against M. abscessus biofilms (Table 2). EC/11716 was also bactericidal against M. abscessus biofilms (Biofilm MBC = 6.3 μM) (Table 2). While the biofilm MBC of EC/11716 increased 2-fold relative to that determined against planktonic bacteria, this compound had better bactericidal activity against biofilms than moxifloxacin (Biofilm MBC = 25 μM). Thus, EC/11716 retained its growth inhibitory activity against M. abscessus biofilms and is more effective against biofilms than moxifloxacin.

Under low oxygen or low nutrient conditions, M. abscessus can enter a nonreplicating state that also allows the bacteria to become tolerant to drugs with bactericidal activity. Given the fact that EC/11716 is bactericidal against replicating, planktonic M. abscessus (Table 2), we asked whether this compound could retain its activity against nonreplicating M. abscessus Bamboo under anaerobic or nutrient-starved conditions. EC/11716 showed no bactericidal activity under these culture conditions (MBCs > 100 μM, Table 2).

### Resistance against EC/11716 maps to M. abscessus DNA gyrase.

Consistent with NBTIs targeting DNA Gyrase, MGI-resistant M. tuberculosis mutants carried point mutations in the enzyme (42). To determine whether DNA gyrase is the target of EC/11716 in M. abscessus, we selected for EC/11716-resistant mutants of M. abscessus Bamboo (Table 3). We calculated the frequency of resistance of M. abscessus to EC/11716 as 1.8 × 10^−8^/CFU, which was similar to that reported for MGIs in M. tuberculosis (7.4 × 10^−8^/CFU) (42). MIC profiling of four resistant mutants (RM1-4) showed high level resistance to EC/11716 (MIC > 100 μM) but no resistance to clarithromycin (Table 3). RM1-4 also showed moderate cross-resistance to fluoroquinolones with a 4 to 8-fold increase in moxifloxacin MICs (Table 3). Sequencing of *gyrA* (*MAB_0019*) and *gyrB* (*MAB_0006*) revealed that all four resistant strains had missense mutations in the hydrophobic pocket that is formed by the GyrA dimer interface and bound by NBTIs/MGIs (Table 3) (35, 36, 42, 55). Two of the mutations we observed (D91N and D91G) were previously reported in MGI-resistant M. tuberculosis mutants (42). In agreement with our results in M. abscessus, the D91N and D91G M. tuberculosis mutants also displayed low level fluoroquinolone cross-resistance (42). These results suggest that EC/11716 exerts its anti-NTM activity by targeting DNA gyrase as previously described for other NBTIs/MGIs (36, 42).

**TABLE 3:** Characterization of M. abscessus EC/11716-resistant mutants^*a*^

	MIC (μM)^*b*^			
**Strain**	**CLR**	**MXF**	**EC/11716**	**GyrA/B mutations**
M. abscessus Bamboo	0.33	5.4	3.6	None
RM1	0.24	20	>100	GyrA M129K
RM2	0.29	42	>100	GyrA D91N
RM3	0.27	47	>100	GyrA D91N
RM4	0.26	27	>100	GyrA D91G

a MIC values are the mean of two independent experiments.

b CLR, clarithromycin; MXF, moxifloxacin.

Due to their different on-target mechanism of action, MGIs retain activity against fluoroquinolone-resistant M. tuberculosis harboring missense mutations in DNA gyrase (42). To determine whether this holds true in M. abscessus, we tested the activity of EC/11716 against three M. abscessus fluoroquinolone-resistance conferring DNA gyrase mutant strains (MXF_R1-3) (Table 4). MXF_R1-3 exhibited high resistance to moxifloxacin (MIC > 50 μM) due to different GyrA QRDR missense mutations at D96 (Table 4). However, all three mutants remained susceptible to EC/11716 (MIC ≤ 10 μM) with one of the strains showing low level cross-resistance (Table 4). Therefore, the limited cross-resistance of fluoroquinolone-resistant M. tuberculosis mutants to MGIs is also observed in M. abscessus.

**TABLE 4:** EC/11716 is active against fluoroquinolone-resistant M. abscessus^*a*^

		MIC (μM)^*b*^		
**Strain**	**GyrA/B mutations**	**CLR**	**MXF**	**EC/11716**
M. abscessus ATCC 19977	None	1.2	3.6	1.9
MXF_R1	GyrA D96Y	1.5	>100	10
MXF_R2	GyrA D96N	0.73	68	2.5
MXF_R3	GyrA D96G	0.34	54	2

a MIC values are the mean of two independent experiments.

b CLR, clarithromycin; MXF, moxifloxacin.

### Pharmacokinetic properties of EC/11716.

**FIG 2:**
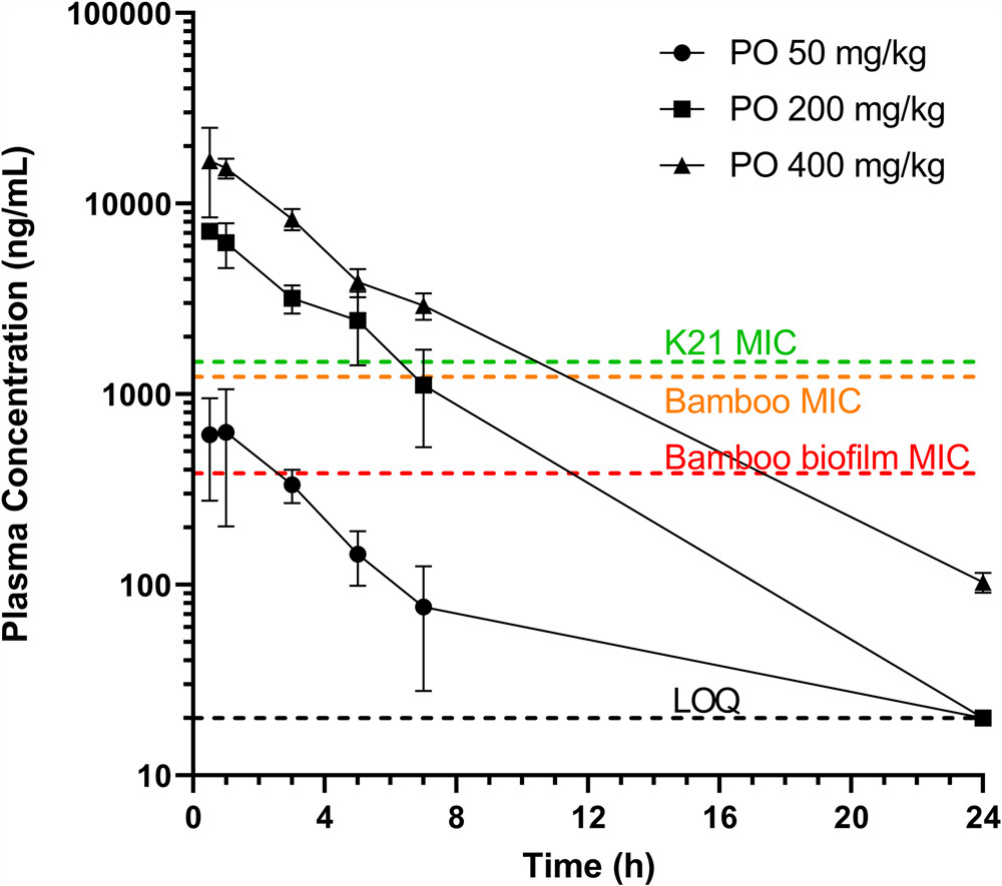
Plasma concentration versus time profiles for EC/11716. Plasma concentrations versus time profiles of EC/11716 after oral (PO) administration of 50, 200, and 400 mg/kg. MIC values of M. abscessus Bamboo and K21 planktonic cultures (Table 2) and M. abscessus Bamboo biofilms (Table 3) are plotted. For the 50 and 200 mg/kg doses, the plasma concentration at the 24h time point was below the limit of quantitation (LOQ, 20 ng/ml).

EC/11716 exhibited attractive physicochemical properties (MW of 494.4 and LogD of 1.74), resulting in moderate permeability and moderate protein binding (Table 5). After oral administration in mice, EC/11716 was rapidly absorbed at all doses tested (50, 200 and 400 mg/kg), consistent with its good solubility and permeability (Fig. 2). *In vivo* clearance was low to moderate with an elimination half-life of 3.6 h (± 0.2 h), consistent with the moderate mouse microsomal clearance of 1.6 ml/min/g protein (Table 5). Human microsomal clearance was significantly lower (<0.3 ml/min/g protein) suggesting translational potential for the compound class (Table 5). Exposure was more than dose proportional, particularly between 50 and 200 mg/kg, as reflected by the dose-normalized exposure measured at the three doses (Table 5, dose-normalized area under the concentration-time curve, or DNAUC). This could be due to saturation of elimination processes at 200 mg/kg, which might be offset by solubility-limited absorption at 400 mg/kg. Using these PK profiles and EC/11716 potency data, we determined that 400 mg/kg would achieve 67% time above MIC of M. abscessus K21 planktonic cultures (Fig. 2 and Table 1). In addition, 400 mg/kg achieved greater than 70% time above MIC of M. abscessus biofilms (Fig. 2 and Table 2). Since EC/11716 was tolerated at doses up to 550 mg/kg, we proceeded with an M. abscessus efficacy study at 400 mg/kg.

**TABLE 5:** Physicochemical and pharmacokinetic properties of EC/11716^*a*^

Property	Value
Physicochemical and *in vitro* pharmacokinetic parameters	
CLND solubility (μM)	525
ChromLogD pH 7.4	1.73
AMP pH 7.4 (nm/sec)	160
Protein binding^*b*^ (%)	49.5
Mouse and human hepatic microsomes stability (*in vitro*)^*c*^	
Mouse *In vitro* CL_int_ (ml/min/g tissue) Human *In vitro* CL_int_ (ml/min/g tissue)	1.6 < 0.3
Mouse oral pharmacokinetic parameters^*d*^	
*C*_max__50 mg/kg (ng/ml) *C*_max__200 mg/kg (ng/ml) *C*_max__400 mg/kg (ng/ml) *T*_max__50 mg/kg (h) *T*_max__200 mg/kg (h) *T*_max__400 mg/kg (h) AUC_[0-24]__50 mg/kg (ng*h/ml) AUC_[0-24]__200 mg/kg (ng*h/ml) AUC_[0-24]__400 mg/kg (ng*h/ml) DNAUC_[0-24]__50 mg/kg (ng*h/ml per mg/kg) DNAUC_[0-24]__200 mg/kg (ng*h/ml per mg/kg) DNAUC_[0-24]__400 mg/kg (ng*h/ml per mg/kg) Elimination *T*_1/2 __50 mg/kg (h) Elimination *T*_1/2 __200 mg/kg (h) Elimination *T*_1/2 __400 mg/kg (h)	632 (430) 7,177 (618) 16,767 (8,281) 0.5–1.0 0.5−1.0 0.5–1.0 2895 (787) 33378 (4,860) 80,663 (3,400) 58 (16) 167 (24) 202 (8) 2.7 (0.3) 1.7 (0.1) 3.6 (0.2)

a CLND solubility, aqueous solubility via chemiluminescent nitrogen detection; AMP, artificial membrane permeability; CL_int_, intrinsic clearance; *C*_max_, peak plasma concentration; *T*_max_, time taken to reach *C*_max_; AUC_[0-24]_, area under the concentration-time curve from 0 to 24h; DNAUC, dose normalized area under the concentration-time curve.

b To human serum albumin.

c Microsomes were generated with the free base form of EC/11716.

d Values are mean (SD). T_max_ is expressed as a range of values.

### EC/11716 is active against M. abscessus *in vivo*.

**FIG 3:**
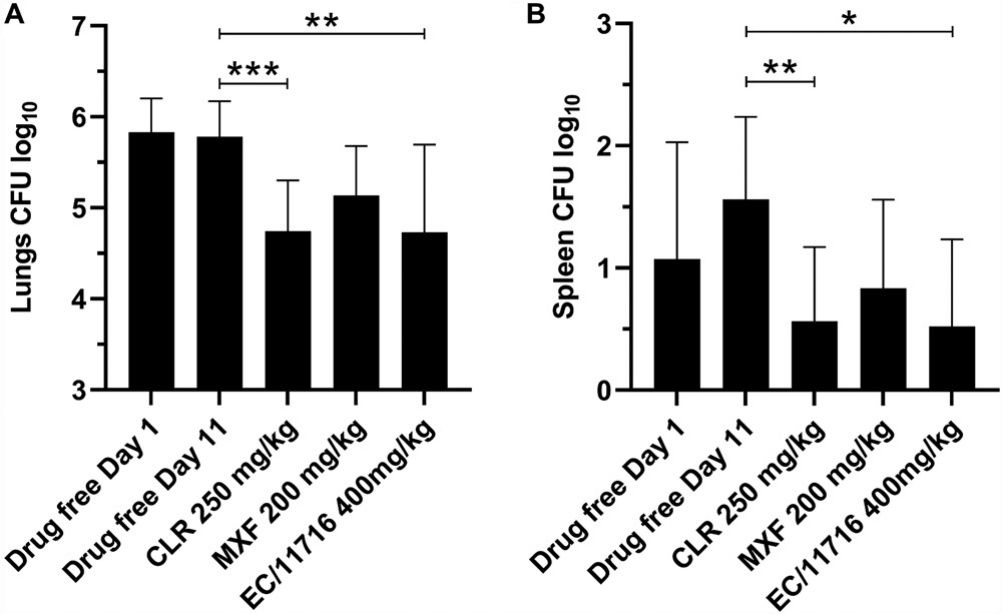
EC/11716 is active against M. abscessus *in vivo*. Lung CFU (A) and Spleen CFU (B) from NOD SCID mice 1 day after intranasal infection with *M. abscessus* (Drug free Day 1) and following daily oral administration of drug free vehicle, clarithromycin (CLR), moxifloxacin (MXF) or EC/11716 for 10 days (Day 11). Data represent the mean plus standard deviation of six mice per treatment group. Statistical significance of the results was analyzed by one-way analysis of variance (ANOVA) multicomparison and Tukey’s posttest (*, *P* < 0.05; **, *P* < 0.01; ***, *P* < 0.001).

To determine whether EC/11716 is active against NTM *in vivo*, we examined whether this compound has activity against M. abscessus in a murine infection model (48). NOD SCID mice were infected intranasally with M. abscessus subsp. *abscessus* K21. On day 1 postinfection, the lung bacterial burden reached 6.8 × 10^5^ CFU (Fig. 3A). Starting on day 1, 400 mg/kg EC/11716, 200 mg/kg moxifloxacin, 250 mg/kg clarithromycin or drug free vehicle was administered orally to mice once daily for 10 days. In mice given the drug free vehicle control, the lung bacterial burden remained unchanged after 10 days (Fig. 3A, Day 11). Treatment with EC/11716 at 400 mg/kg achieved a statistically significant 1-log reduction in lung CFU that was on par with clarithromycin dosed at 250 mg/kg (Fig. 3A). In contrast, treatment with moxifloxacin at 200 mg/kg achieved a 0.6-log reduction in lung CFU burden that was not statistically significant (Fig. 3A). We observed a similar trend in CFU reduction in the spleen (Fig. 3B). Therefore, EC/11716 showed efficacy against M. abscessus in a preclinical animal infection model.

## DISCUSSION

The mycobacterial DNA gyrase was first validated as an attractive drug target by fluoroquinolones, which are efficacious against drug-resistant TB. But with the use of fluoroquinolones for NTM lung disease being curtailed by intrinsic drug resistance, it is evident that a novel nonfluoroquinolone gyrase inhibitor would be a valuable addition to the dwindling NTM drug arsenal. For this reason, we tested the mycobacterial gyrase inhibitor (MGI) EC/11716, an advanced TB-active compound from the new class of novel bacterial topoisomerase inhibitor (NBTI)-type DNA gyrase inhibitors. We found that EC/11716 was not only active against M. tuberculosis but also against M. avium and M. abscessus. In the M. abscessus complex, EC/11716’s activity covered all subspecies, was bactericidal and effective against biofilms. Furthermore, EC/11716 was active in a M. abscessus mouse infection model, providing *in vivo* proof-of-concept for this novel NTM active. Thus, EC/11716 validates an approach for *de novo* NTM drug discovery starting from advanced compounds with TB activity, allowing rapid progression from hit to a lead with demonstrated efficacy (56, 57).

Our study represents the first report of an MGI/NBTI with anti-NTM activity. Selection of M. abscessus EC/11716-resistant strains with GyrA mutations confirmed DNA Gyrase as the target of the compound in NTM. In addition, all of the observed resistance mutations are located in the pocket of the GyrA dimer interface that is targeted by MGIs. RM1 carried a mutation at M127, a residue that lines the hydrophobic pocket and makes van der Waals interactions with MGIs (35, 36, 55). The other three mutants (RM2-4) had mutations at D91, which sits at the entrance to the pocket and makes a hydrogen bonding interaction with MGI scaffolds (35, 36, 42, 55). As observed in our M. abscessus mutants, these DNA gyrase mutations affect MGI potency but confer only moderate fluoroquinolone cross-resistance in other bacteria (35, 42). Thus, not only the target but also the unique on-target mechanism of NBTIs appears to be conserved in NTM.

In conclusion, we examined whether MGIs, a subclass of TB active NBTI DNA gyrase inhibitors, have NTM activity by testing an advanced compound from this series. We found that EC/11716 is an anti-NTM compound, introducing MGIs as a new compound class for NTM drug discovery. EC/11716’s bactericidal and anti-biofilm properties, combined with acceptable pharmacokinetic and efficacy data, establish this compound as an attractive preclinical anti-NTM lead compound.

## MATERIALS AND METHODS

### Bacterial strains, culture media, and drugs.

M. abscessus Bamboo was isolated from the sputum of a patient with amyotrophic lateral sclerosis and bronchiectasis and was provided by Wei Chang Huang, Taichung Veterans General Hospital, Taichung, Taiwan. M. abscessus Bamboo whole-genome sequencing showed that the strain belongs to M. abscessus subsp. *abscessus* and harbors an inactive clarithromycin-sensitive *erm*(41) C28 sequevar (43, 58). M. avium 11 was isolated from the bone marrow of a patient with AIDS with disseminated infection and was provided by Jung-Yien Chien and Po-Ren Hsueh, National Taiwan University Hospital, Taipei. Whole-genome sequencing showed that the strain belongs to M. avium subsp. *hominissuis* (44).

Mycobacterium abscessus subsp. *abscessus* ATCC 19977, harboring the inducible clarithromycin resistance-conferring *erm*(41) T28 sequevar (59), was purchased from the American Type Culture Collection (ATCC). Mycobacterium abscessus subsp. *bolletii* CCUG 50184T, harboring the inducible clarithromycin resistance-conferring *erm*(41) T28 sequevar (60), and Mycobacterium abscessus subsp. *massiliense* CCUG 48898T, harboring the nonfunctional *erm*(41) deletion sequevar (61), were purchased from the Culture Collection University of Goteborg (CCUG). M. tuberculosis H37Rv ATCC 27294 and M. intracellulare ATCC 13950 were purchased from the ATCC.

Clinical isolates covering the M. abscessus complex (M9, M199, M337, M404, M422, M232, M506, M111) were provided by Jeanette W. P. Teo (Department of Laboratory Medicine, National University Hospital, Singapore). The subspecies and *erm*(41) sequevars of these isolates were determined previously (47). M. abscessus subsp. *abscessus* K21 was isolated from a patient and provided by Sung Jae Shin (Department of Microbiology, Yonsei University College of Medicine, Seoul, South Korea) and Won-Jung Koh (Division of Pulmonary and Critical Care Medicine, Samsung Medical Center, Seoul, South Korea). This strain harbors the inactive, clarithromycin-sensitive *erm*(41) C28 sequevar as determined previously (48).

For general bacteria culturing and certain MIC experiments, Middlebrook 7H9 broth (BD Difco) supplemented with 0.5% albumin, 0.2% glucose, 0.085% sodium chloride, 0.0003% catalase, 0.2% glycerol, and 0.05% Tween 80 was used. Unless otherwise stated, solid cultures were grown on Middlebrook 7H10 agar (BD Difco) supplemented with 0.5% albumin, 0.2% glucose, 0.085% sodium chloride, 0.5% glycerol, 0.0003% catalase, and 0.006% oleic acid. Cation-adjusted Mueller-Hinton (CAMH) broth was prepared by first preparing Mueller-Hinton broth (Oxoid CM0405) according to the manufacturer’s instructions and then supplementing aseptically with sterile solutions of CaCl_2_ and MgSO_4_ to achieve CLSI recommended divalent cation levels (Ca^2+^, 25 mg/liter; Mg^2+^, 12.5 mg/liter).

EC/11716 was provided by GlaxoSmithKline. The synthesis of EC/11716 is described in patents WO 2010081874 A1 (Example 5, Page 40) and US 2012/0115899 A1 (Example 5, Page 22). Clarithromycin and moxifloxacin were purchased from Sigma-Aldrich (C9742 and SML1581, respectively). For *in vitro* studies, all drugs were prepared as 10 mM stocks in 100% DMSO.

### MIC assay in 96-well plate format.

Unless otherwise stated, MIC determination was carried out in 96-well plate format as previously described (47, 56). 96-well plates were initially set up with 100 μl of 7H9 per well. For each compound, a 10-point 2-fold dilution series starting at twice the desired highest concentration was dispensed onto the 96-well plates using a Tecan D300e Digital Dispenser, with the DMSO concentration normalized to 2%. A bacterial culture grown to midlog phase (OD_600_ =0.4–0.6) was diluted to OD_600_ = 0.1 (1 × 10^7^ CFU/ml). 100 μl of the resulting bacteria suspension was dispensed onto the 96-well plates containing compounds to give a final volume of 200 μl per well with an initial OD_600_ = 0.05 (5 × 10^6^ CFU/ml) and final DMSO concentration of 1%. Final compound concentration ranges were typically 50–0.098 μM or 6.25–0.012 μM but were adjusted to 100–0.195 μM for testing of EC/11716-resistant mutant strains. Untreated control wells are included on each plate that contain bacteria suspension and 1% DMSO. Plates were sealed with parafilm, stored in boxes with wet paper towels and incubated at 37°C with shaking (110 rpm). Plates were incubated for 3 days (M. abscessus complex), 4 days (M. avium complex) or 7 days (M. tuberculosis). To determine growth, OD_600_ was measured using a Tecan Infinite M200 plate reader on day 0 and day 3, 4 or 7. Two biological replicates were performed. Clarithromycin (M. abscessus and M. avium complexes) or Rifampin (M. tuberculosis) were included in each experiment as a positive control.

For each well on the 96-well plate, bacterial growth was calculated by subtracting the day 0 OD_600_ value from the endpoint (day 3, 4 or 7) OD_600_ value. For each compound series, the bacterial growth values for the untreated control wells were averaged to give the average drug-free bacterial growth. For compound-containing wells, percentage growth was calculated by dividing their growth values by the average drug-free bacterial growth for the compound series and multiplying by 100. For each compound series, we plotted percentage growth versus compound concentration. By visual inspection of the dose-response curve, we determined the MIC of a compound as the compound concentrations that would result in 90% growth inhibition.

For MIC Determination in CAMH broth, experiments were set up as described above with the following changes. Compounds were dispensed onto 96-well plates with 100 μl of CAMH broth per well. A midlog phase bacterial culture (initially in 7H9) was washed once and resuspended with CAMH broth. The culture was then diluted to OD_600_ = 0.1 (1 × 10^7^ CFU/ml) using CAMH broth before dispensing to the 96-well plates.

### MIC and MBC determination in culture tubes.

M. abscessus Bamboo culture was grown to midlog phase (OD_600_ =0.4–0.6) and diluted to OD_600_ = 0.1 (1 × 10^7^ CFU/ml). 1.2 ml aliquots of the resulting bacteria suspension were transferred into 14 ml vented, round-bottom tubes (catalog no. 150268; Thermo Fisher, Rochester, NY, United States). A 10-point 2-fold dilution series of the compound was prepared, starting at 100 times the desired highest concentration. The compound concentration range tested was100–0.195 μM. For each drug concentration tested, 12 μl of drug stock was added to two tubes and vortexed. 12 μl of DMSO was added to two tubes as the untreated control (1% final DMSO concentration). From each tube, 200 μl was transferred to wells on a 96-well plate and the OD_600_ was measured using a Tecan Infinite M200 plate reader (day 0 reading). The tubes (1 ml final volume) were incubated on a tilted rack at 37°C on an orbital shaker at 220 rpm. On day 2, tubes were vortexed before transferring 200 μl onto a 96-well plate to measure the OD_600_ again (day 2 reading). To determine the MIC, day 0 and day 2 OD_600_ values were analyzed as previously described for MIC determination in 96-well plate format. To determine the MBC, CFU measurement was done for the OD_600_ = 0.1 bacteria suspension on day 0 and for each tube on day 2. Specifically, serial 10-fold dilutions were prepared in phosphate-buffered saline (Thermo Fisher catalog no. 10010023) containing 0.025% Tween 80 (PBS/Tween 80) and plated on 7H10 agar. The MBC was defined as the lowest concentration of drug that reduced the CFU/ml value by 10-fold relative to the day 0 CFU/ml.

### Biofilm growth inhibition assay.

The biofilm growth inhibition assay was performed as previously described (52). Innovotech MBEC 96-well Biofilm Assay Plates (Innovotech 19111, Edmonton, AB, Alberta, Canada) were used, and the supplier’s manual was followed with minor modifications. Midlog phase M. abscessus Bamboo precultures (OD_600_ = 0.4–0.6) were spun down at 3,200 × *g* for 10 min at 25°C and washed with 7H9 medium without Tween 80 (7H9nt). Bacteria were resuspended into 25 ml 7H9nt to an OD_600_ = 0.0125 (1.0 × 10^6^ CFU/ml). 150 μl of bacteria suspension was dispensed into each well of MBEC multititer plates and the polystyrene protrusions (pegs) of the MBEC lid were inserted into the culture-containing wells for 24 h at 37°C on an orbital shaker at 110 rpm to allow attachment of the bacteria to the pegs and initiation of biofilm growth. The lids with the pegs (now with 1-day-old biofilms) were transferred to new MBEC multititer plates containing 150 μl of fresh 7H9nt medium per well without bacteria (0 h time point). After that, the lids with biofilm-laden pegs were transferred once a day to a new multititer plate containing fresh 7H9nt medium. To measure growth of the biofilm formed on the peg, the pegs were washed in 200 μl of 7H9nt medium before they were aseptically removed and placed in 1.7 ml microcentrifuge tubes (VWR 87003-294, Rador, PA, United States) containing 500 μl PBS/Tween 80. The microcentrifuge tubes were vigorously vortexed at 2,000 rpm for 90 s at 25°C to detach the bacteria from the pegs before samples were serially diluted and plated for the determination of CFU/peg. To determine the biofilm MIC of antibiotics, the daily transfers of the MBEC plate lids from the 24 h time point onwards were to new MBEC plates with 150 μl 7H9 media containing appropriate drug concentrations or DMSO (untreated control, 1% final concentration), and CFU (on the pegs) were determined after 48 h of incubation with antibiotic (72 h time point). The average drug-free biofilm growth was calculated by subtracting the average 24 h CFU/peg value from the average 72 h CFU/peg value for the untreated control pegs. The biofilm MIC was defined as the lowest drug concentration that reduced the CFU/peg by 90% relative to the average drug-free biofilm growth.

The biofilm MBC was defined as the concentration of drug that reduced the CFU/peg by 10-fold relative to the CFU/peg at 24 h.

### MBC determination under nutrient-starved conditions.

The determination of drug bactericidal activity against nutrient-starved M. abscessus cultures was performed as previously described (52). Midlog phase M. abscessus Bamboo precultures (OD_600_ = 0.4–0.6) were spun down at 3200 × *g* for 10 min at 25°C and washed three times with phosphate-buffered saline containing 0.025% Tyloxapol (Sigma-Aldrich T0307-10G, St. Louis, MO, United States) (PBS/Tyloxapol). Washed culture was then diluted with PBS/Tyloxapol to OD_600_ = 0.2 (1.8 × 10^7^ CFU/ml). One hundred milliliter of OD_600_ = 0.2 cultures were incubated in roller bottles (Corning 430195, Oneonta, NY, United States) at 37°C and 2 rpm, and 0 h time point CFU were determined by plating on 7H10 agar. At 144 h post-incubation, CFU were determined again and 1 ml aliquots of the roller bottle cultures were transferred into 14 ml vented, round-bottom tubes (Thermo Fisher 150268, Rochester, NY, United States). Appropriate concentrations of drugs up to 100 μM were added to the tubes prior to incubation at 37°C on a tilted rack in an orbital shaker at 220 rpm. After 48 h of incubation with antibiotics (192 h time point) CFU were enumerated. The MBC was defined as the lowest concentration of drug that reduced the 192 h CFU/ml value by 10-fold relative to the 144 h CFU/ml value.

### MBC determination under anaerobic conditions.

The determination of drug bactericidal activity against anaerobic M. abscessus cultures was performed as previously described (52). Midlog phase M. abscessus Bamboo precultures (OD_600_ = 0.4–0.6) were diluted with fresh 7H9 medium to OD_600_ = 0.02. 7 ml aliquots were transferred to 10 ml air-tight vacutainer tubes (BD 366430, Franklin Lakes, NJ, United States) containing elliptical stir bars (Radleys RR98096, Wood Dale, IL, United States). Tubes were incubated at 37°C on magnetic stirring platforms at 150 rpm. To monitor depletion of oxygen, methylene blue was added to cultures at a concentration of 1.5 μg/ml. The dye decolorized after 5 days (120 h time point), indicating anaerobiosis. At the 144-h time point, CFU was determined, and appropriate concentrations of drugs up to 100 μM were added using a 23G needle (BD 305194, Franklin Lakes, NJ, United States) to minimize the reintroduction of air after achieving anaerobiosis. Cultures were then incubated for 48 h with antibiotics before plating and CFU enumeration (192 h time point). The MBC was defined as the lowest concentration of drug that reduced the 192 h CFU/ml value by 10-fold relative to the 144 h CFU/ml value.

### Selection of spontaneous resistant mutants.

Spontaneous resistant mutants were selected as described previously (62). For EC/11716, exponentially growing M. abscessus Bamboo culture (10^7^ to 10^9^ CFU) was plated on 7H10 agar containing 100 μM drug. (62). For moxifloxacin, exponentially growing M. abscessus subsp. *abscessus* ATCC 19977 culture (10^7^ to 10^9^ CFU) was plated on 7H10 agar containing either 192 or 384 μM drug. The plates were incubated for 7 days at 37°C. Apparent resistant colonies were purified and confirmed by restreaking on agar containing the same concentration of EC/11716 or moxifloxacin. Genomic DNA was extracted as described previously using the phenol-chloroform method (63). Sanger sequencing of the *gyrA* (*MAB_0019*) genomic region was performed by Genewiz (GENEWIZ, Inc., South Plainfield, NJ, USA; www.genewiz.com) using four primers (GyrA_F1, 5′-GCATCTAAAGCCGCTGAGAACG-3′; GyrA_R1, 5′-GAGGTTGTTCAGCACCACCTTGG-3′; GyrA_F2, 5′-GCGGGCATCTCCAACATCGAGG-3′; GyrA_R2, 5′-GGTCCACGGGGCGTTCGTTTGC-3′). Sanger sequencing of the *gyrB* (*MAB_0006*) genomic region was performed by Genewiz using four primers (gyrB_F1na, 5′-GGCGTGGTGACGAGTTTAAAG-3′; gyrB_F2na, 5′-GAGATCTTCGAGACCACCACCTA-3′; gyrB_F3na, 5′-GCAAGAGTGCCACCGATATC-3′; gyrB_R1na, 5′-GTAAGTACGACGGCACAACG-3′).

### Kinetic aqueous solubility assay.

The aqueous solubility of test compounds was measured using an in-house method utilizing quantification via chemiluminescent nitrogen detection (CLND): 5 μl of 10 mM DMSO stock solution was diluted to 100 μl with pH 7.4 phosphate-buffered saline and equilibrated for 1 h at RT, filtered through Millipore Multiscreen HTS-PCF filter plates (MSSL BPC). The eluent is quantified by a suitably calibrated flow injection CLND (or CAD). This assay has a dynamic range between the lower detection limit of 1 and 500 μM, governed by the protocol’s 1:20 dilution into pH 7.4 phosphate buffer solution from nominal 10 mM DMSO stock.

### ChromlogD assay.

The Chromatographic Hydrophobicity Index (CHI) values are measured using reversed phase HPLC column (50 × 2 mm 3 μM Gemini NX C18, Phenomenex, UK) with fast acetonitrile gradient at starting mobile phase of pHs 2, 7.4 and 10.5. CHI values are derived directly from the gradient retention times by using a calibration line obtained for standard compounds. The CHI value approximates to the volume % organic concentration when the compound elutes. CHI is linearly transformed into ChromlogD by least-square fitting of experimental CHI values to calculated ClogP values for over 20K research compounds using the following formula: ChromlogD = 0.0857CHI-2.00. The average error of the assay is ±3 CHI unit or ±0.25 ChromlogD.

### AMP (artificial membrane permeability) assay.

An 8% L-a-phosphatidylcholine (EPC) in 1% cholesterol decane solution and a 1.8% EPC in cholesterol decane solution were prepared. The lipid solution was aliquoted into 4 ml capped vials, sealed with parafilm and stored in −20°C freezer. The lipid solution was then transferred from 4 ml vial into a 96-well half area plate (130 μl/well) for daily assay usage. An additional 50 mM phosphate buffer with 0.5% encapsin, pH at 7.4 was prepared. The assay was run by the Biomek FX and Biomek software. The assay procedure is written under the Biomek software. For one batch assay, it can test two 96-well sample plates with at least one standard on each sample plate. The total assay time was about 4 h. 3.5 μl of lipid solution were added to the filler plate, shaken for 12 s, and 250 μl of buffer were added to donor side and 100 μl to the receiver side. The assay plate was shaken for 45 min before adding the compounds. The test compounds (2.5 μl) were added to the donor side. The assay was run as replicates: Assay plates 1 and 2 tested the sample plate 1; assay plates 3 and 4 tested the sample plate 2. The assay plates were then incubated and shaken for 3 h at room temperature. The assay samples were transferred to the HPLC analysis plates and 100 μl of receiver solution were aspirated and transferred to the receiver for analysis. Similarly, another 100 μl from the donor solution were transferred to the donor analysis plate. Compound concentration was measured by HPLC at different time points.

### Drug binding to human serum albumin.

The determination of drug binding to human serum albumin (HSA) was carried out by retention time measurements using immobilized HSA HPLC columns obtained from Chiral Technologies Ltd., France. The column dimensions were 50 × 3 mm. The mobile phase was 50 mM ammonium acetate buffer pH 7.4 and HPLC grade 2-propanol. HPLC Method: Flow rate 1.8 ml/min applying 2.5 min 2-propanol gradient up to 30%. From 2.5 min to 4.5 min the 2-propanol concentration in the mobile phase was kept at 30%. From 4.5 min to 4.6 min, the 2-propanol concentration was decreased to 0% and kept like that until the end of the gradient run which was 6 min. A calibration set of compounds was analyzed first for which plasma protein binding data were available: Warfarin, Nizatidine, Bromazepam, Carbamazepine, Budesonide, Nicardipine, Indomethacine Piroxicam, Naproxen.

### Stability in microsomes.

Intrinsic clearance (CLi) values were determined in mouse and human liver microsomes. Test compounds (final concentration 0.5 μM) were incubated at 37°C for 30 min in 50 mM potassium phosphate buffer (pH 7.4) containing 0.5 mg microsomal protein/ml. The reaction was started by addition of cofactor NADPH. At 10 different time points (from zero to 30 min) an aliquot (90 μl) was taken, quenched with acetonitrile-methanol containing an appropriate internal standard, centrifuged and analyzed by LC-MS/MS. The intrinsic clearance (CLi) was determined using the following equation:

CLi=k*(ml of incubation/mg microsomal protein)*(mg microsomal protein/g liver)

Where k is the turn-over rate constant of the ln (% remaining compound) versus time regression and mg microsomal protein/g liver is 52.5 for both mouse and human.

### Pharmacokinetics studies.

All animal studies were ethically reviewed and carried out in accordance with the Institutional Animal Care and Use Committee of Hackensack Meridian Health. Six-week-old CD-1 female mice (20–25 g) were used in pharmacokinetic studies. Groups of 3 mice received a single oral dose of EC/11716 formulated in 0.4% methylcellulose, at either 50, 200 or 400 mg/kg. Aliquots of 20 μl of blood were collected from the lateral tail vein by serial puncture from each mouse at 0.5, 1, 3, 5, 7 and 24h post dose in K_2_EDTA tubes. Plasma was recovered after centrifugation and stored at −80°C until analyzed by high pressure liquid chromatography coupled to tandem mass spectrometry.

### LC-MS/MS analytical methods.

Neat 1 mg/ml DMSO stocks of EC/11716 were serial diluted in 50/50 Acetonitrile water to create standard curves and quality control spiking solutions. Standards and QCs were created by adding 10μls of spiking solutions to 90μls of drug free plasma (CD-1 K2EDTA Mouse, Bioreclamation IVT). 10μls of control, standard, QC, or study sample were added to 100 μl of Acetonitrile/Methanol 50/50 protein precipitation solvent containing 10 ng/ml of the internal standard Verapamil (Sigma-Aldrich). Extracts were vortexed for 5 min and centrifuged at 4000 RPM for 5 min. 75 μl of supernatant was transferred for HPLC-MS/MS analysis and diluted with 75 μl of Milli-Q deionized water.

LC-MS/MS analysis was performed on a Sciex Applied Biosystems Qtrap 6500+ triple-quadrupole mass spectrometer coupled to a Shimadzu Nexera X2 UHPLC system to quantify each drug in plasma. Chromatography was performed on a Phenomenex Luna Omega column (2.1 × 100 mm; particle size, 3 μm) using a reverse phase gradient. Milli-Q deionized water with 0.1% formic acid was used for the aqueous mobile phase and 0.1% formic acid in acetonitrile for the organic mobile phase. Multiple-reaction monitoring of parent/daughter transitions in electrospray positive-ionization mode was used to quantify the analytes. The following MRM transitions were used for EC/11716 (458.09/203.10) and Verapamil (455.40/165.00). Sample analysis was accepted if the concentrations of the quality control samples were within 20% of the nominal concentration. Data processing was performed using Analyst software (version 1.6.2; Applied Biosystems Sciex).

### M. abscessus mouse infection model.

*In vivo* efficacy determinations were carried out as described previously, using 8-week-old female NOD.CB17-*Prkdc^scid^*/NCrCrl (NOD SCID) mice (Charles River Laboratories) and the M. abscessus subsp. *abscessus* K21 strain (48). Briefly, anesthetized animals were infected by intranasal delivery of ∼10^6^ CFU of M. abscessus subsp. *abscessus* K21. Acute infection was achieved within 1 day. Drugs or the vehicle control was administered to NOD SCID mice once daily for 10 consecutive days by oral gavage, starting from 1 day postinfection. Clarithromycin was formulated in 0.4% methyl cellulose–sterile water and administered at a dose of 250 mg/kg. Moxifloxacin was formulated in sterile water and administered at 200 mg/kg. EC/11716 was formulated in 0.4% methyl cellulose–sterile water and administered at 400 mg/kg. All mice were euthanized 24 h after the last dose (11 days postinfection), and their lungs and spleen were aseptically removed prior to homogenization. The bacterial load in these organs was determined by plating serial dilutions of the organ homogenates onto Middlebrook 7H11 agar (BD Difco) supplemented with 0.2% (vol/vol) glycerol and 10% (vol/vol) OADC. The agar plates were incubated for 5 days at 37°C prior to counting of colonies. All studies were conducted in accordance with the GSK Policy on the Care, Welfare and Treatment of Laboratory Animals and were reviewed the Institutional Animal Care and Use Committee either at GSK or by the ethical review process at the institution where the work was performed. All experiments involving live animals were approved by the Institutional Animal Care and Use Committee of the Center for Discovery and Innovation, Hackensack Meridian Health.
